# Endophytic fungal community structure in olive orchards with high and low incidence of olive anthracnose

**DOI:** 10.1038/s41598-020-79962-z

**Published:** 2021-01-12

**Authors:** Fátima Martins, Diogo Mina, José Alberto Pereira, Paula Baptista

**Affiliations:** 1 Centro de Investigação de Montanha (CIMO), Instituto Politécnico de Bragança, Campus de Santa Apolónia, 5300-253 Bragança, Portugal; 2 Department of Engineering and Agricultural Science, University of Leon, Avda. Portugal No. 41, 24071 León, Spain

**Keywords:** Microbial ecology, Microbial communities, Pathogens, Fungi, Fungal ecology

## Abstract

Fungal endophytes have been increasingly recognized to promote host plant protection to pathogens, but knowledge of the multiple effects that they could have in crop diseases is still scarce. This work attempts to understand the role of fungal endophytes in crop diseases, specifically in reducing disease development and interfering on lifestyle transition of the pathogen. To accomplish this, the endophytic fungal community of reproductive organs of olive tree from two orchards showing different levels of anthracnose incidence, a major disease of olive fruits, was characterized and compared between them. The two orchards showed distinct endophytic communities, differing in species richness, abundance and composition, with highest isolation rates and richness of endophytes in the orchard with low anthracnose incidence. These differences among orchards were greater on fruits than on flowers, suggesting that these changes in endophytic fungal composition may influence the lifestyle shifts in pathogen (from latent to pathogen). A number of fungal taxa were found to be positively associated to one of the two orchards. The fungal endophytes best correlated with high incidence of anthracnose are pathogens, while endophytes-associated to low anthracnose incidence are described to protect plants. Altogether, the results suggest varying pathogen–endophyte interactions among the two orchards.

## Introduction

In nature, every plant species has been found to cohabit with a great diversity of endophytic microorganisms, including fungi^1^. The members of these complex fungal communities continually interact among each other and with their hosts, sometimes conferring benefits (mutualistic) and at other times causing harm (pathogenic) by contributing to disease in plants^2^. However, the importance of multispecies interaction on plant diseases has been mostly recognized from studies involving pathogen–pathogen interaction^3,4^. In contrast, the relevance for plant health of the interaction between the pathogen and other microbial groups associated to plants is still scarce^3^. Mounting evidence shows that specific microbial taxa that co-occur with the pathogen may impact the pathogenic process^5,6^. For example, some non-pathogenic bacteria that co-occur with the pathogen *Pseudomonas savastanoi* pv. *savastanoi* (Psv) have been showed to increase the severity of olive knot disease^7,8^. Besides bacteria, also fungi have been recently suggested to interact with Psv in olive tree twigs with important implications for the development of the olive knot disease^9^. In contrast, no clear interaction between naturally endophytes and the pathogen *Xylella fastidiosa* was observed in symptomless infected olive tree^10^. These studies reveal how complex and contradictory is the significance of pathogen–microbial interaction for plant health^5^.

Other challenging question that may have significant implications on pathogenesis is related to the pathogen–microbial interaction importance in the change of the pathogen’s lifestyle. Most phytopathogenic fungi start their lifecycle with a quiescent phase, remaining unnoticed, before switching to an active necrotrophic lifestyle in which the disease symptoms became visible^11^. Studies addressing the factors underlying this switch of lifestyle have been only focused on host plant (*e.g*., physiological, chemical and defense response), pathogen (*e.g*., virulence genes, effectors) or environmental aspects^12^, completely disregarding the plant microbiota. The potential importance that this microbial community might have in maintaining or facilitating the pathogen transition from quiescence to necrotrophic colonization is still unknown.

The anthracnose, caused by several species of the *Colletotrichum* genus, is considered to be one of the most globally-distributed and economically-important disease, responsible for significant losses in many crops^13^, including olive^14^. In the olive tree, this disease affects mostly the fruits, especially when they are nearly ripened^14^. Several species of *Colletotrichum* spp. responsible to cause olive anthracnose have been reported to exhibited different lifestyles, ranging from latent infections, at the flowering stage, to a necrotrophic phase upon fruit ripening^15^. Before this devastating stage these fungi can also adopt endophytic- or hemibiotrophic-like lifestyle before fruit ripening^13^. These characteristics make the olive tree and the anthracnose disease a good model system for studying the influence of the plant microbiota on the outcome of plant–pathogen interactions.

In light of the above, this work compared the endophytic fungal communities of flower buds, flowers and fruits of olive tree between orchards with high and low anthracnose incidence, in order to elucidate the potential role of plant microbiota on the pathogenesis process. Specifically, we want to answer the following questions: (i) In what way do the endophytic fungal communities differ between orchards with different disease incidence? (ii) In what way do the endophytic fungal communities differ among the different pathogen’s lifestyle (*i.e*., from early flowering stages until fruit set)? (iii) Is there any fungal consortium specifically associated with high (“disease-promoting fungi”) or low (“disease-suppression fungi”) anthracnose incidence as well as with a specific pathogen’s lifestyle? A better knowledge of the importance of plant fungal endophytes on the anthracnose incidence may help to develop holistic management strategies against this disease. In this work, was used a cultivable approach to evaluate the endophytic fungal community, which allows the further study of the functions and application of the isolates identify to limit or prevent anthracnose disease.

## Results

### *Anthracnose incidence and* Colletotrichum *abundance*

**Figure 1:**
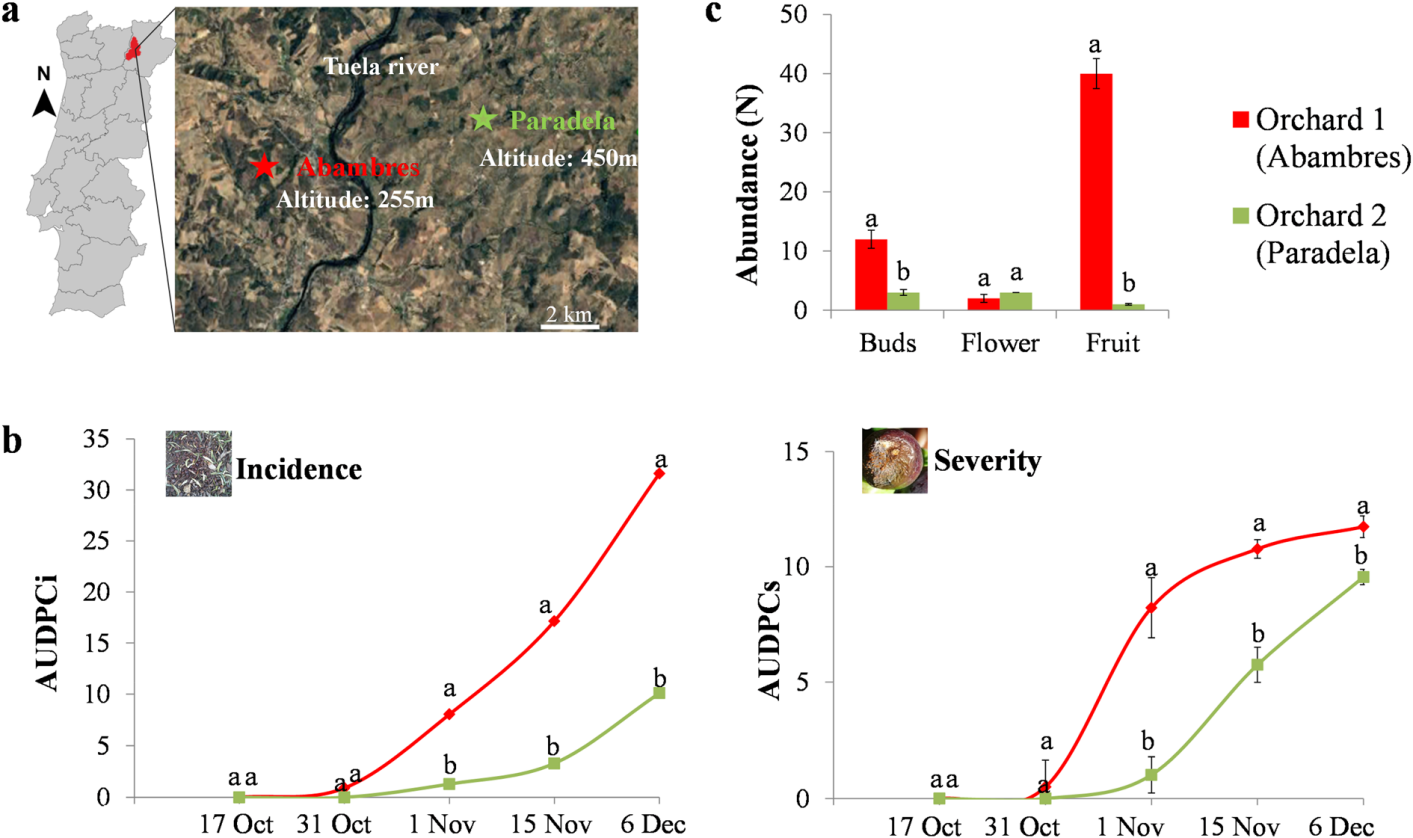
Olive orchards used for the isolation of endophytic fungi from flower buds, flowers and fruits of olive tree. (**a**) Google map showing the localization of the two olive orchards—orchard 1 (Abambres) and 2 (Paradela) in the Northeast of Portugal (retrieved on 10th December 2019, from https://www.google.pt/maps/); (**b**) Area under the disease progress curve of incidence (AUDPCi) and severity (AUDPCs) of olive anthracnose evaluated in both orchards during the autumn–winter period of 2016; (**c**) Abundance of *Colletotrichum* spp. in flower buds, flowers and fruits of olive trees from the two orchards. Data points with different letters indicate statistically significant differences (at least *p* < 0.05) between the two olive orchards.

In this study was evaluated the endophytic fungal community associated to olive trees from two orchards with a different historical record of anthracnose incidence. The results from disease progression curves also clearly differentiated the two orchards regarding the levels of anthracnose (Fig. 1b). Indeed, both disease incidence (AUDPCi) and severity (AUDPCs) were significantly (*p* < 0.001) higher in the orchard with a history of anthracnose (orchard 1—Abambres; AUDPCi = 8.0 and AUDPCs = 8.2) than in the orchard with a historically low incidence of anthracnose (orchard 2—Paradela; AUDPCi = 1.3 and AUDPCs = 1.0). These differences were noticed after the 1st November and becoming higher for incidence over time as fruit matured. For simplification, the orchard with high and low incidence of anthracnose will be termed from now as “HighI” and “LowI”, respectively.

The isolation of *Colletotrichum* spp. from the interior of asymptomatic reproductive organs of trees located in both orchards, also confirms the high prevalence of the pathogen in the orchard with high incidence (Fig. 1c). Indeed, the abundance of *Colletotrichum* spp. in flower buds and fruits was significantly higher (up to 4.0-fold and 40.0-fold, respectively, *p* < 0.001) in the orchard with HighI when compared to the orchard with LowI. More importantly, the results also showed the ability of these *Colletotrichum* isolates to grow asymptomatically inside the host in an endophytic and/or quiescent manner. The identified endophytic *Colletotrichum* strains belonging to *C. acutatum* J.H. Simmonds complex—*C. godetiae* Neerg., which was found in all organs surveyed, and *C. fioriniae* (Marcelino & Gouli) Pennycook, that was detected in both flowers and fruits (data not showed).

### Fungal community comparison: high versus low anthracnose incidence

Analysis of fungal communities in olive trees from the two orchards revealed a total of 115 operational taxonomic units (OTUs), belonging to two phyla, 31 families and 61 genera. Ascomycota was the most abundant phylum, accounting for 96% of the total isolates and the remaining isolates belonged to Basidiomycota (Fig. S1). *Biscogniauxia* (Xylariaceae), *Colletotrichum* (Glomerellaceae), *Alternaria* (Pleosporaceae) and *Cladosporium* (Cladosporiaceae) were the most abundant genera, accounting together with 44% of total isolates.

**Figure 2:**
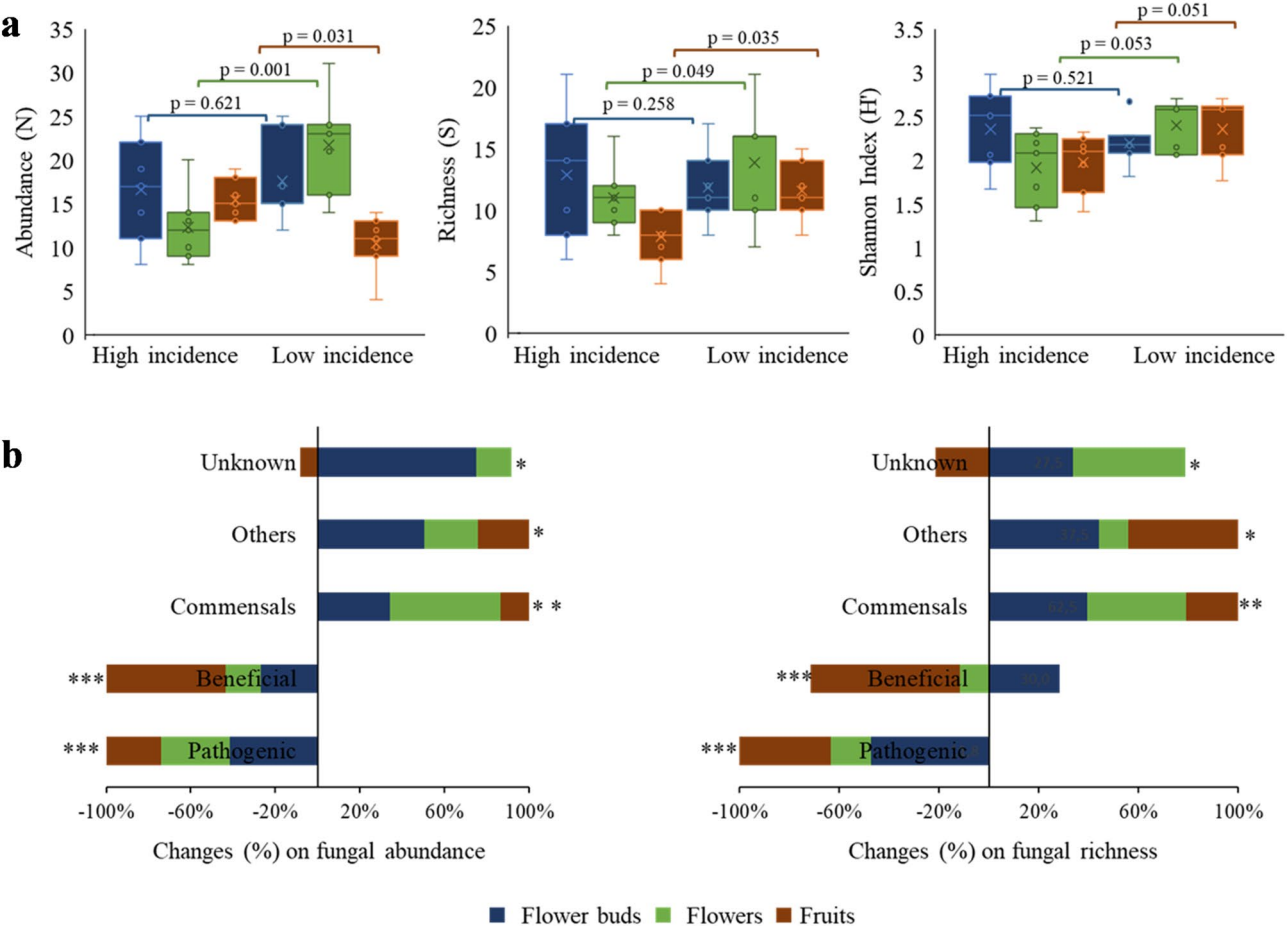
Comparison of endophytic fungal diversity in reproductive organs of olive tree between orchards with high (High incidence) and low (Low incidence) incidence of olive anthracnose. (**a**) Diversity at community level by determining abundance, richness and Shannon–Wiener index. Box plots depict medians (central horizontal lines), the inter-quartile ranges (boxes), 95% confidence intervals (whiskers), and outliers (dots). Statistically differences between pairs of values are showed over horizontal lines. (**b**) Changes (%) on endophytic fungal abundance and richness between orchards with high and low incidence of anthracnose for each functional group. Asterisks indicate statistically significant differences between the two orchards (*p < 0.05, **p < 0.01, ***p < 0.001).

The endophytic fungal community associated with olive tree varied between the two different orchards (*i.e*., HighI and LowI). Overall, there was a significantly greater abundance (up to 1.3-fold; *p* = 0.001) and richness (up to 1.2–fold; *p* = 0.05) of fungal endophytes in the orchard with low versus high anthracnose incidence, while Shannon–Wiener diversity (*p* = 0.05) were similar among orchards (Fig. 2a). The increase in fungal abundance was more evident within endophytes of flowers (1.8-fold increase, *p* = 0.001); whereas an opposite result was observed in fruits (1.5-fold decrease, *p* = 0.031). Similarly, fungal richness in flowers and fruits was up to 1.5-fold and 1.3-fold significantly (*p* < 0.05) higher in the orchard with LowI than in the ones with HighI, respectively.

The fungal functional categories that changed more between the two orchards were beneficial and pathogenic fungi, and in less extent commensals (Fig. 2b). A significant decline in abundance and richness of beneficial and pathogenic fungi was observed in the orchard with HighI in relation to the orchard with LowI. This decrease was most notorious within the endophytes colonizing the fruits (for beneficials) and the flower buds (for pathogens). Commensals increased significantly their abundance and richness in the orchard with LowI in relation to the orchard with HighI, in particular within endophytes inhabiting flower buds and flowers.

**Figure 3:**
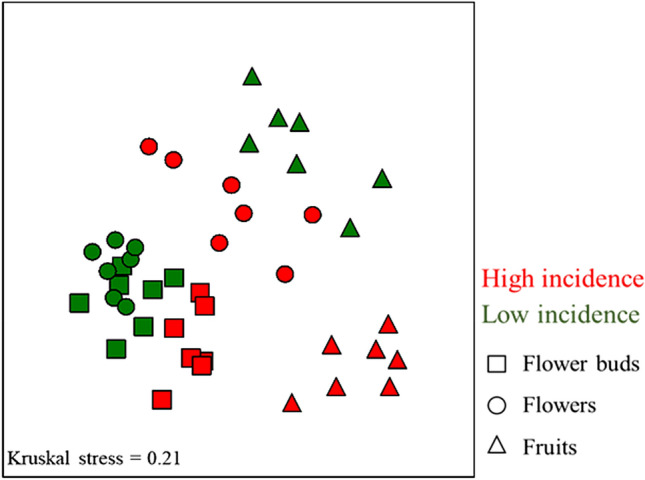
Non-metric multidimensional scale (NMDS) plots for the endophytic fungal assemblages in olive tree due to different olive orchards (with high or low incidence of olive anthracnose) and organ (flower buds, flowers and fruits). Cluster analysis was performed with Bray–Curtis coefficient (raw abundance data). Kruskal’s stress values are presented (values less than 0.2 represent good ordination plots).

The whole fungal community composition significantly differs between orchards with high and low incidence of anthracnose, as revealed by the non-metric multidimensional scaling (NMDS) plots and analysis of similarities (ANOSIM; Global R = 0.76, *p* = 0.001) based on Bray–Curtis index (Fig. 3). These differences were higher within endophytes of fruits (R = 0.91, *p* = 0.001) than of flowers (R = 0.74, *p* = 0.001) or flower buds (R = 0.55, *p* = 0.001). For each orchard was similarly found differences on fungal endophytic composition between the three olive tree organs (Fig. 3), being these differences slightly greater in the orchard with HighI (Global R = 0.77, *p* = 0.001) than in the orchard with LowI (Global R = 0.70, *p* = 0.001). Pairwise comparisons performed in orchards with high and low anthracnose incidence indicated that the fungal composition in the flower buds and flowers were the most similar (R = 0.61 and R = 0.60, *p* = 0.001, respectively), followed by the flower buds and fruits (R = 0.90 and R = 0.92, *p* = 0.001, respectively) and flowers and fruits (R = 0.91 and R = 0.95, *p* = 0.001, respectively).

**Figure 4:**
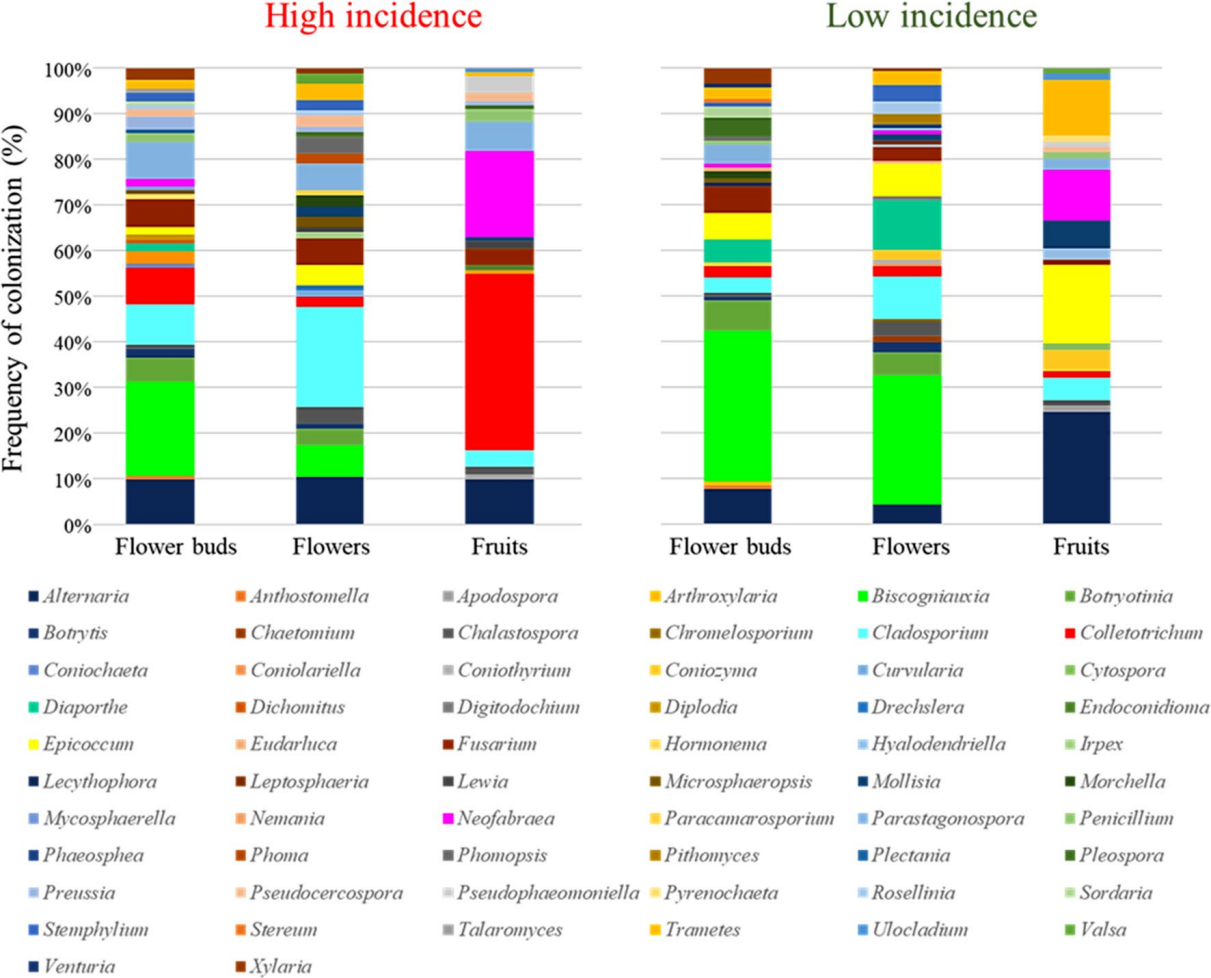
Frequency of colonization (%) of fungal endophytes in flower buds, flowers and fruits of olive tree growing in orchards with high (High incidence) and low (Low incidence) incidence of olive anthracnose.

The trees growing in the orchard with HighI were colonized mostly by *Biscogniauxia*, *Cladosporium* and *Colletotrichum*, infecting more than 1.4, 1.6 and 2.6% of the total flower buds, flowers and fruits segments surveyed, respectively (Fig. 4). The most dominant fungi in trees from orchard with LowI were *Biscogniauxia*, which occurred on more than 1.9% and 1.6% of the flower buds and flowers analyzed, respectively, and *Alternaria* that colonized more than 1.2% of the fruits. Likewise, each orchard had several OTUs that were unique: 37 fungal OTUs were isolated only in the orchard with HighI and 33 were recovered only in the orchard with LowI (Fig. S2).

### Contribution of different drivers for fungal community shaping

The relative contribution of the type of olive orchard (*i.e*., HighI and LowI) and plant organ (flower buds, flowers and fruits) to the assembly of endophytic fungal community was assessed by using a variation partitioning (*Varpart*) analysis. Results revealed that fungal composition was mainly explained by the plant organ (responsible for 24% of the total variation, F = 2.17, *p* = 0.005), while the type of orchard explained 10% of the total community variation (F = 1.98, *p* = 0.005). A random forest analysis was then performed to identify which fungal OTUs were most important in differentiating either orchards (*i.e*., with high and low anthracnose incidence; Fig. S3) or olive tree organs (*i.e*., flower buds, flowers and fruits; Fig. S4). This analysis identified a total of ten and seventeen different fungal OTUs as being important in distinguishing the two olive orchards (Fig. S3) and plant organs (Fig. S4), respectively. The fungal OTUs that most differentiate orchards were *Epicoccum nigrum* Link (isolated from flowers and fruits), *Biscogniauxia mediterranea* (De Not.) Kuntze and *Diaporthe rudis* (Fr.) Nitschke (both isolated from flower buds and flowers) (Fig. S3). Plant organs were distinguished mostly by the fungal OTUs *B. mediterranea* (in both HighI and LowI orchards) as well as *Neofabraea vagabunda* (Desm.) Rossman and *Pezizomycetes* sp. isolated in olive orchards with HighI and LowI, respectively (Fig. S4).

### Fungal OTUs associated with each olive orchard and plant organ

**Figure 5:**
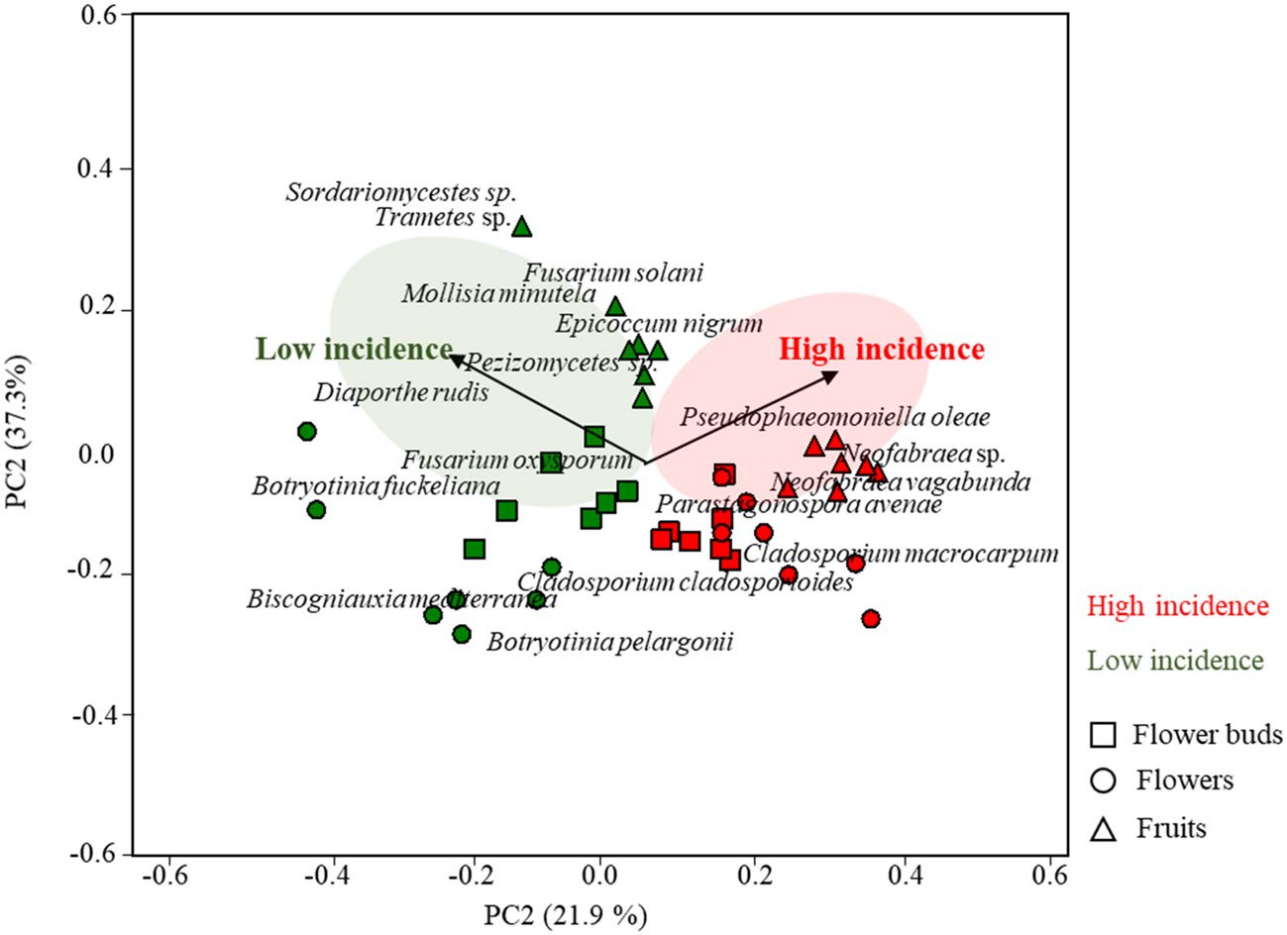
Principal component analysis (PCA) of endophytic fungal community inhabiting flower buds, flowers and fruits of olive tree growing in orchards with high incidence (High incidence) and low (Low incidence) incidence of olive anthracnose. This analysis was performed with preselected fungal OTUs by the random forest analysis.

In our study, the results show that both type of olive orchard and plant organ play an important role in fungal community assembly, suggesting the existence of an endophytic consortium associated with each orchard or plant organ. With an attempt to identify the composition of such consortium, a PCA was performed by using the fungal OTUs previously selected by the random forest analysis (Figs. S3 and S4). Results revealed that the fungal OTUs *Pseudophaeomoniella oleae* Nigro, Antelmi & Crous, *N. vagabunda*, *Neofabraea* sp. and *Parastagonospora avenae* (A.B. Frank) Quaedvl., Verkley & Crous, were the most associated with high anthracnose incidence, being all of them isolated from fruits (Fig. 5). This result is corroborated by the significantly positive correlation found between these fungal OTUs with *Colletotrichum* spp. abundance (Table S1). In contrast, *Diaporthe rudis* (Fr.) Nitschke (from flowers), *Fusarium* aff. *oxysporum* Schltdl. (from flower buds), *Pezizomycetes* sp., *E. nigrum*, *Mollisia minutella* (Batsch) P. Karst., *Trametes* sp. and *Sardariomycetes* sp. (all isolated from fruits) were found to be highly associated to orchard with low anthracnose incidence (Fig. 5). Some of these fungal OTUs were also found to be significantly negatively correlated with *Colletotrichum* spp. abundance, as well as many other fungal OTUs (Table S1).

## Discussion

In the present work, the diversity and composition of endophytic fungi of reproductive organs of olive tree from olive orchards with HighI and LowI of anthracnose was investigated. Differences between these two orchards on disease incidence and severity as well as on *Colletotrichum* inoculum levels were corroborate by field anthracnose disease assessments, which validate their suitability to investigate the relevance of the endophytic fungal communities of olive tree on pathogen pressure. The olive cultivar surveyed was the same in both orchards, but probably there could be some differences in the environmental conditions due to the higher proximity of the orchard 1 (Abambres) to the river when compared to orchard 2 (Paradela). Thus, it is likely that the relative humidity, which is a very important factor for anthracnose development^16^, can be slightly higher on orchard 1 than on orchard 2. This hypothesis needs to be confirmed. Apart from differences on *Colletotrichum* abundance and probably on relative humidity values, both orchards are within the same edapho-climatic zone and are managed in the same way.

In this study the endophytic fungal community associated to reproductive organs of olive tree growing in the two orchards was evaluated by using a culture-dependent method. Such approach may introduce biasness in estimating fungal diversity, being recently deduced a ratio of 1:8.8 of the numbers of cultured fungi and the OTUs detected by different culture-independent methods^17^. However, culture-independent techniques may also have disadvantages. For example, most of the fungal OTUs identified by this technique were only assignable to order, family or genus level^18,19^. In the culture-dependent method, most of the fungal taxa can be identify to species level^19^. Our priority in this work was to identify the wide variety of fungal taxa at the species level and get the cultures of specimens, in order to study in future works their role in the pathogenesis of the *Colletotrichum* spp., the causative agent of olive anthracnose. Although there are several growth media for cultivating fungal endophytes^20^, we chose to use basic nutrient-rich media (PDA), so that the beneficial endophytes could be easily mass-produced for agriculture use. Although the limitation of culture-dependent methods in recovering a small portion of the diversity^17^, most of the fungal OTUs detected in the present work belong to Sordariomycetes and Dothideomycetes classes, as previously reported by Abdelfattah et al.^21^ who studied the fungal community associated to flowers and fruits of olive tree by 454-pyrosequencing.

The endophyte communities of reproductive organs of olive tree at the two orchards were distinct, differing in species richness, abundance and composition. Although this variation between orchards did not provide information regarding the pathogen cause and effect, these results suggest varying pathogen–fungal community interactions in the orchard with high and low disease incidence. Perhaps, in the olive orchard with LowI both the pathogens *Colletotrichum* spp., and the disease cannot thrive so well as in the orchard with HighI due to the richer and more abundant endophyte community. Indeed, in many cases the tolerance of host plants to diseases was correlated to increased endophytic fungal and/or bacterial diversity^22,23^. For example, the resistance of the olive cultivar “Leccino” towards the pathogen *Xylella fastidiosa* was recently ascribed to their diverse endophytic communities and the presence of cultivar-specific bacterial taxa that appear to interact with the pathogen^23^. Additionally, in the orchard with HighI was observed a decline in abundance and richness of beneficial endophytic fungi, which may probably contain antagonistic fungi that limit the development of *Colletotrichum* spp.. and of disease. Some of the declined fungal OTUs we identified have previously been reported to inhibit *Colletotrichum* spp. in detached olive fruits, such as *E. nigrum* and *Chaetomium globosum* Kunze^24^. Similarly, many other studies have already showed the capacity of fungal or bacterial endophytes to interact directly with the pathogen in a way that can affect the progress of the disease in several pathosystems^22,23^. Besides beneficial fungi, the abundance and richness of pathogenic fungi of other plant species was also observed to decline in the olive orchard with HighI. These results suggest that this group of fungi might also have an important role in the development of olive anthracnose. Indeed, the interspecific competition between pathogenic fungi colonizing various plant parts has been already showed to play an important role in pathogenesis^4^. Besides *Colletotrichum*-endophytic interaction, the differences on weather conditions that probably occur among olive orchards might not be disregarded as a valid explanation for the differences in anthracnose incidence. Indeed, we cannot rule out that the historical record of high anthracnose incidence in one of the orchards surveyed may be due to the more favorable weather conditions for pathogen infection than in the orchard with low anthracnose incidence. Thus, differences in the endophyte fungal communities among orchards do not really imply they cause differences in anthracnose incidence. The weather conditions may also be an important causal factor. Accordingly, the context-dependency in *Colletotrichum*–endophytic interaction seems to be the most reasonable reason for the different anthracnose rates observed among orchards. There has been little work studying the context-dependency of endophyte-mediated disease outcomes in field/realistic conditions^25^. The few studies available in controlled conditions showed that plant–pathogen–endophyte interaction outcomes are dependent on the environmental conditions^25^, reinforcing our assumption.

*Colletotrichum* species responsible to cause olive anthracnose may be either commensal or pathogenic depending on the developmental stage of the host^15^. During the flowering, the fungus adopts an endophytic- or latent-lifestyle, and upon fruit ripening the fungus shift to a necrotrophic phase^13,14^. Changes on endophytic species composition among orchards were mostly noticed in fruits than in flowers, suggesting that these changes may influence the lifestyle shifts in *Colletotrichum* spp.. The transition of the fungus *Moniliophthora perniciosa* (Stahel) Aime & Phillips-Mora to a more aggressive lifestyle was previously suggested to be triggered by their interaction with other plant microbes^26^. Our hypothesis is reinforced by the fact that a higher difference on fungal endophytic composition among flowers and fruits is observed at the orchard with the higher anthracnose incidence. Thus, it seems that there is a relationship between the fungal community structure and anthracnose disease incidence: the greater similarity on fungal composition between fruits and flowers (where *Colletotrichum* spp. occurs as latent), the lesser is disease incidence. Such hypothesis should be confirmed in future work. Furthermore, it is also important to determine whether anthracnose fruit incidence is affected by the level of latent *Colletotrichum* spp. inoculum in the flowers. Since no acervuli/conidial masses was observed on flowers colonized by *Colletotrichum* spp. during the whole work, we predict that these tissues may act as a reservoir for fruit rot epidemics that occur in the autumn. However, this has yet to be confirmed under field conditions.

Plant organ was also found to significantly affect the composition of endophytic fungal community in the olive tree endosphere as reported previously for several woody plant species^27^, including olive tree^28,29^. However, most of these studies have been focused on vegetative organs, being less studied the microbiota that inhabits the reproductive organs of woody species. As far as we known, only Abdelfattah et al.^21^ have similarly found differences on fungal composition (both epiphytes and endophyte) between fruits and flowers of olive tree. The organ-specificity of fungal endophytes observed in our work could be related to morphological and chemical differences between flowers and fruits, as previously suggested for vegetative organs^29^. Flowers and fruits seem to produce distinct microhabitats, potentially selecting for specific microbial colonizers. Since the switching of *Colletotrichum* spp. from endophytic to pathogenic mode occur preferably on fruits, we hypothesized that the changes in endophytic community composition between flowers and fruits may play an important role in the anthracnose development. Thus, the ability of each plant organ to shape its endophytic community seems to serve as an additional layer of defense to *Colletotrichum* spp.. Indeed, flower microbiota has been increasingly recognized as important agents of disease control^30^. Such hypothesis needs however to be confirmed.

Our data revealed that a consortium of fungal OTUs is associated with each organ in orchards with different anthracnose incidence. It is assumed that these consortia probably have relevance to the pathogenesis of *Colletotrichum* spp. and consequently to disease incidence. The fungal OTUs best correlated with high incidence of anthracnose are pathogens of olive tree (*Neofabraea* sp., *N. vagabunda* and *P. oleae*)^31,32^ or of wheat plants (*P. avenae*)^33^. These fungi are positively correlated with *Colletotrichum* abundance, and can act as “pathogen facilitators”, helping the pathogen to successfully infect the plant or increase the severity of the disease. In contrast, *D. rudis* (from flowers), *F.* aff. *oxysporum* (from flower buds), *Pezizomycetes* sp., *E. nigrum*, *M. minutella*, *Trametes* sp. and *Sardariomycetes* sp. (all isolated from fruits) were found to be highly associated to orchard with low anthracnose incidence. Most of these fungal OTUs have been reported as protective. For example, *E. nigrum* isolated from rye and wheat grains demonstrated to be effective against *Fusarium* spp., limiting its growth under in vitro conditions^34^. The capacity of non-pathogenic strains of *F. oxysporum* to control several fungal diseases has been also demonstrated^35^. Species of the genus *Trametes* are well known for their ability to produce enzymes, which could assist in the defense of the plant against pathogens^36^. Similarly, species from Pezizomycetes have been reported to offer benefits to their host plants^37^.

In summary, the abundance, richness and composition of fungal endophytes inhabiting the reproductive organs of olive tree differ between orchards with variable anthracnose incidence, suggesting the variation of *Colletotrichum*–endophytic interactions in the orchard with high and low disease incidence. These differences were greater on fruits than on flowers, which suggested that the lifestyle transition in *Colletotrichum* spp. from latent (during flower stage) to pathogen (during fruit stage) might be related to the endophytic fungal community structure. A set of fungal OTUs were found to be associated to orchards with either high or low anthracnose incidence, which their role to decrease (antagonism) or increase (facilitation) olive anthracnose development should be studied in a near future.

## Materials and methods

### Study area and plant material collection

The plant material was collected from June to December 2016 in two olive orchards, one historically with high anthracnose incidence (orchard 1—41° 33 45"N, 7° 10′ 58"W) and the other with low incidence of anthracnose (orchard 2—41° 33 08″N, 7° 07′ 24″W), located in the same region (*i.e*., Mirandela, Northeast of Portugal) (Fig. 1a). Their selection was based on the local knowledge of Association of Producers in Integrated Pest Management of Trás-os-Montes and Alto Douro region (APPITAD), who has a deep knowledge of local olive groves. These two olive orchards were the most similar in terms of edaphoclimatic conditions and management practices in the whole region, but with contrasting levels of anthracnose incidence. These differences between the two orchards were confirmed by estimating the disease incidence and severity simultaneously to sample collection (please see anthracnose incidence and severity section). Both orchards are located ≈15 km apart, but in areas with different altitude (Fig. 1a). The first orchard is located in Abambres at 255 m altitude and at ≈ 2 km from the river, while the second orchard is located in Paradela at 450 m altitude (≈ 8 km from the river). Both orchards are composed of olive trees from cv. *Madural* (which is moderately susceptible to anthracnose), with a similar age (< 30 years old), planted at 7 × 7 m spacing and managed through integrated production guidelines^38^. From each orchard, seven olive trees were randomly selected, and asymptomatically flower buds, flowers and fruits were collected around the perimeter of the tree at the operator height and used for isolation of fungal endophytes. The sampling was conduct in several dates spanning the entire period from flower bud until fruit set. The plant material collected were divided into three groups: (1) flower buds, which comprised different development stages (*i.e*., petals just visible, green petals longer than sepals and petals whitening); (2) flowers having different stages of development (*i.e*., on flower cluster development, and on flower cluster totally expanded, flowers open and petals fallen or faded); (3) fruits, which were picked at veraison (fruit matures from yellow–green) and mature (fruit skin turns from purple to black and the flesh darkens). In total, 50 samples of each flower buds, flowers and fruits, were collected per tree, being each tree corresponding to a biological replicate. The samples collected were placed directly into sterile bags, transported to the laboratory and stored at 4 °C until isolation of endophytic fungi, which was carried out within 72 h.

### Anthracnose incidence and severity

Both incidence (*i.e*. percentage of infected fruits) and severity (*i.e*. proportion of fruit area that is affected) of anthracnose were assessed in the same trees used to isolated fungal endophytes. This was done simultaneously to olive fruits collection, at five different dates, from October–December 2016. At each time, was randomly collected a total of 100 fruits *per* tree around the canopy at 1.5–2 m above ground height and placed directly into sterile bags. The fruits were transported to the laboratory in an icebox, and after 1 week of incubation in a wet chamber (80% relative humidity, 22 °C) the fruits were examined for the appearance of symptoms. The disease incidence was determined by the percentage of infected fruits, and severity was determined by using a 0 to 5 rating scale, where 0 = no visible symptoms, 1 = visible symptoms affecting < 25% of the fruit surface, 2 = 25–49%, 3 = 50–74%, 4 = 75–100%, and 5 = soapy fruit^39^. The area under disease progress curve for disease incidence (AUDPCi) and severity (AUDPCs) was further calculated for each tree and sampling date, following the procedure described by Moral et al.^39^.

### Endophytic fungi isolation

The collected plant tissues were briefly washed under distilled water and further subjected to surface sterilization through sequential immersion in a series of solutions as follows: sodium hypochlorite 3% (v/v) for 1 min (for flower buds and flowers) or 2 min (for fruits), 70% (v/v) ethanol for 1 min, and sterile distilled water (three times, 1 min each). The sterilized flower buds, flowers and fruits were cut by surgical blade into 2–4 mm segments and plated onto Difco potato dextrose agar (PDA) medium supplemented with 0.01% (w/v) chloramphenicol (Oxoid, Basingstoke, Hampshire, UK). The success of surface sterilization method was confirmed by imprinting the surface of the plant segments onto PDA medium. In total 4200 segments of each flower buds, flowers and fruits were inoculated (2 orchards × 7 olive trees × 50 plant tissues × 6 tissues segments). The plates were incubated at 25 ± 2 °C in the dark and were daily observed to check the growth of endophytic fungi from the plant segments. All endophytic fungal isolates obtained were purified through single spore isolation technique in fresh PDA medium for later identification.

### Identification of endophytic fungi

Endophytic fungi were first grouped into morphotypes according to their morphological features (*i.e*., colony appearances, characteristics of the hyphae, spores and reproductive structures). After that, two representative isolates of each morphotype were selected for molecular identification, using the internal transcribed spacer (ITS) region of the nuclear ribosomal DNA (rDNA). The genomic DNA was extracted from fungal spores/mycelium, using the REDExtract-N-Amp Plant PCR kit (Sigma, Poole, UK) following the manufacturer’s instructions. The PCR reactions, with the pair of primers ITS1 and ITS4^40^ were performed using the same DNA extraction kit following the manufacturer’s instructions, in the MyCycler thermal cycler (BioRad). The temperature cycle used in the amplification was 94° C for 3 min (1 cycle); 94° C for 30 s, 53° C for 50 s, 72° C for 2 min (35 cycles); and 72° C for 10 min (1 cycle). The amplified products (~ 650 bp) were purified and sequenced using Macrogen Inc. services (Madrid, Spain). The obtained DNA sequences were analyzed with DNASTAR v.2.58 (Lasergene) software, and fungal identification was performed using the NCBI (http://www.ncbi.nlm.nih.gov) and UNITE (https://unite.ut.ee/) databases and BLAST algorithm. The blast results were analyzed based on Raja et al.^41^. Briefly, in the BLAST search was used a query coverage of ≥ 80% and ≥ 96–100% sequence similarity for assigning a species name in one of the following categories: (i) positive species identification for sequence similarity of 100%; (ii) possibly this species (suffix cf.) for sequence similarity of 99%; and (iii) certainly not this species, but taxonomic closely related (suffix aff.) for sequence similarity of 96–98%. Taxonomic classification at genus level was adopted when equal BLAST top score similarity values (ranging from 96 to 100%) were obtained for different species of the same genus or BLAST top score inferior to 96%, but with several species belonging to the same genus. All operational taxonomic units (OTUs) were taxonomically classified at species or genus level according to the Index Fungorum Database (www.indexfungorum.org). For preservation purpose, all isolates identified were deposited in the culture collection of the Mountain Research Centre (CIMO), School of Agriculture—Polytechnic Institute of Bragança.

### Diversity and structure of fungal community

The diversity and composition of endophytic fungal community associated to olive tree was compared between orchards (with high and low incidence of olive anthracnose) or plant organs (flower buds, flowers, and fruits). In this analysis, the *Colletrotrichum* was excluded from the database in order to reflect the true fungal community changes (*i.e*., that is not due to an overabundance of the pathogen in the orchard with high anthracnose incidence). Fungal diversity was assessed by evaluating the abundance (average number of isolates *per* tree) and richness (average number of OTUs *per* tree), and by computing Shannon–Wiener (H’) diversity index with Species Diversity and Richness v. 4.0^42^. The results are presented as the mean of replicates (N = 7), displaying respective SE values. To determine differences among the means, a one-way analysis of variance (ANOVA) with SPSS v.18 software was done, and the averages were compared using Tukey’s test (*p* < 0.05).

Non-metric multidimensional scaling (NMDS) was carried out with Bray–Curtis index, in order to assess the similarity of endophytic assemblages with respect to orchards (with high and low incidence of olive anthracnose) and plant organ (flower buds, flowers, and fruits). Significant differences between fungal community groupings obtained in NMDS ordination was assessed by a one-way analysis of similarity (ANOSIM), using Bray–Curtis distance matrices (obtained from raw abundance data). ANOSIM generates an R-value ranging from 0 (completely similar) to 1 (completely dissimilar) and a *p* value (significant level below 0.05)^43^. Both NMDS and ANOSIM were performed using Community Analysis Package v.5.0^44^.

Composition of fungal community was also compared at functional level. For this, the identified fungal OTUs were firstly classified into functional categories according to Hardoim et al.^1^ and based on literature. This classification included commensal (fungi with not apparent effect on host plants), beneficial (fungi that confers host plant protection and promotes plant growth) and pathogenic (that included latent pathogens) fungal groups. Fungal OTUs belonging to other groups or not able to be identified into a functional group were categorized as “other” and “unknown fungi”, respectively. After grouping, the changes on fungal abundance and richness of each functional group across plant organs were determined among orchards (*i.e*., with high and low anthracnose incidence). A one-way analysis of variance (ANOVA) was performed as previously described, to determine differences on fungal functional groups among orchards.

### Factors driving fungal community structure

To estimate the relative contribution of the type of olive orchard (*i.e*., high and low anthracnose incidence) and plant organ (flower buds, flowers and fruits) in shaping the endophytic community composition a variation partitioning (*Varpart*) analysis was performed with the vegan package of R software using *varpart* function^45^. The significance of each fraction was tested using the *anova.cca* function.

### Identification of fungal OTUs associated with each orchard or plant organ

Random forest analysis was performed to identify which fungal OTUs were the most important in differentiating orchards (high vs. low incidence of anthracnose) or olive tree organs (flower buds, flowers, and fruits). The importance of OTUs to distinguishing fungal community was measure by considering the decrease in mean Gini: a higher decrease will imply a higher importance^46^. This analysis was computed with the *RandomForest* package^47^ from R^45^. The fungal OTUs that were identified to be highly relevant to differentiated orchards or plant organs were selected and subject to principal component analysis (PCA). The PCA aimed to identify the fungal OTUs consortium associated to each orchard and plant organ. This analysis was performed by using the *psych* package^48^ from R^45^. Spearman correlations were also performed in the R *corrplot*^49^ package to assess the correlation between the fungal OTUs preselected by the random forest with the relative abundance of *Colletotrichum* spp.

## Supplementary Information

Supplementary Infomation
