# Supplementary material for: Endophytic fungal community structure in olive orchards with high and low incidence of olive anthracnose
Source: Sci Rep. 2021 Jan 12;11:689. doi: 10.1038/s41598-020-79962-z (PMC7804420; doi:10.1038/s41598-020-79962-z)
Supplement: Supplementary file 1 — Supplementary Infomation [file 41598_2020_79962_MOESM1_ESM.docx]

**Supplementary information**

**Endophytic fungal community structure in olive orchards with high and low incidence of olive anthracnose**

Fátima Martins^1, 2^, Diogo Mina^1^, José Alberto Pereira^1^, Paula Baptista^1^*

^1^Centro de Investigação de Montanha (CIMO), Instituto Politécnico de Bragança, Campus de Santa Apolónia, 5300-253 Bragança, Portugal

^2^Department of Engineering and Agricultural Science - University of Leon, Avda. Portugal nº 41, 24071, León, Spain

* Correspondence:

Paula Baptista

pbaptista@ipb.pt

## Supplementary Figures

**
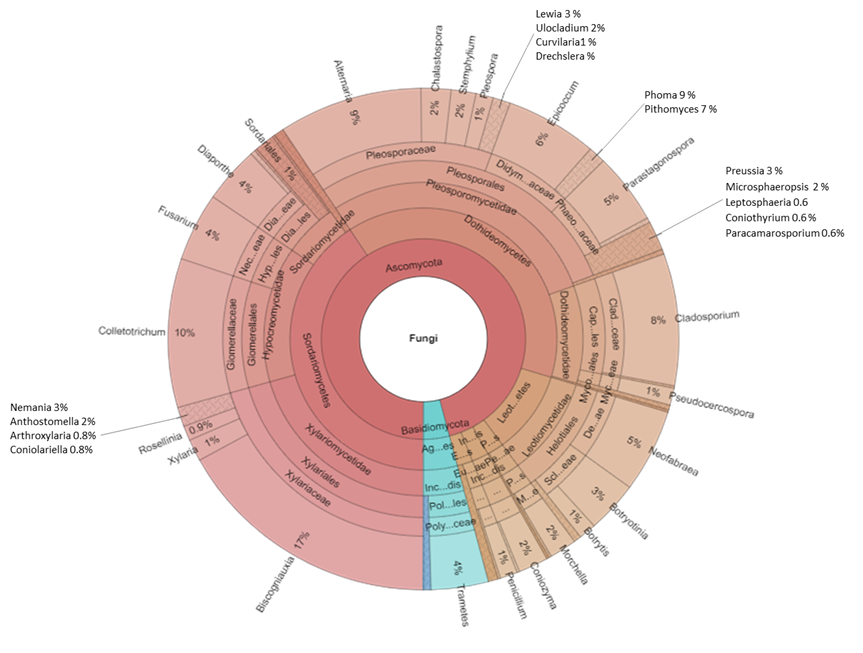
**

**Figure S1.** Krona chart of the taxonomic affiliation down to the genus level of the endophytic fungal community associated to flower buds, flowers and fruits of olive trees from the two orchards surveyed. Figure was constructed using Krona tool^1^.

**Figure S2.** Venn diagrams representing the total number of fungal operational taxonomic units shared between high (High incidence) and low (Low incidence) incidence of olive anthracnose when considering [A] flower buds, [B] flowers and [C] fruits samples.

**Figure S3.** Ranking of the relative importance of each fungal operational taxonomic units (OTUs) to distinguish between the high and low incidence of olive anthracnose in each olive tree organ (flower buds, flowers and fruits). Mean Decrease Gini value measure the importance of OTUs, with highest values representing the best predictors. The OTUs in bold were considered as the main relevant to distinguish olive orchards with high and low incidence of olive anthracnose.

**Figure S4.** Ranking of the relative importance of each fungal operational taxonomic units (OTUs) to distinguish between the olive organs (flower buds, flowers, and fruits) within each olive orchard, high (High incidence) and low (Low incidence) incidence of olive anthracnose. Mean Decrease Gini value measure the importance of OTUs, with the highest values representing the best predictors. The OTUs in bold were considered as the main relevant to distinguish the olive tree organs in each orchard (high and low incidence of olive anthracnose
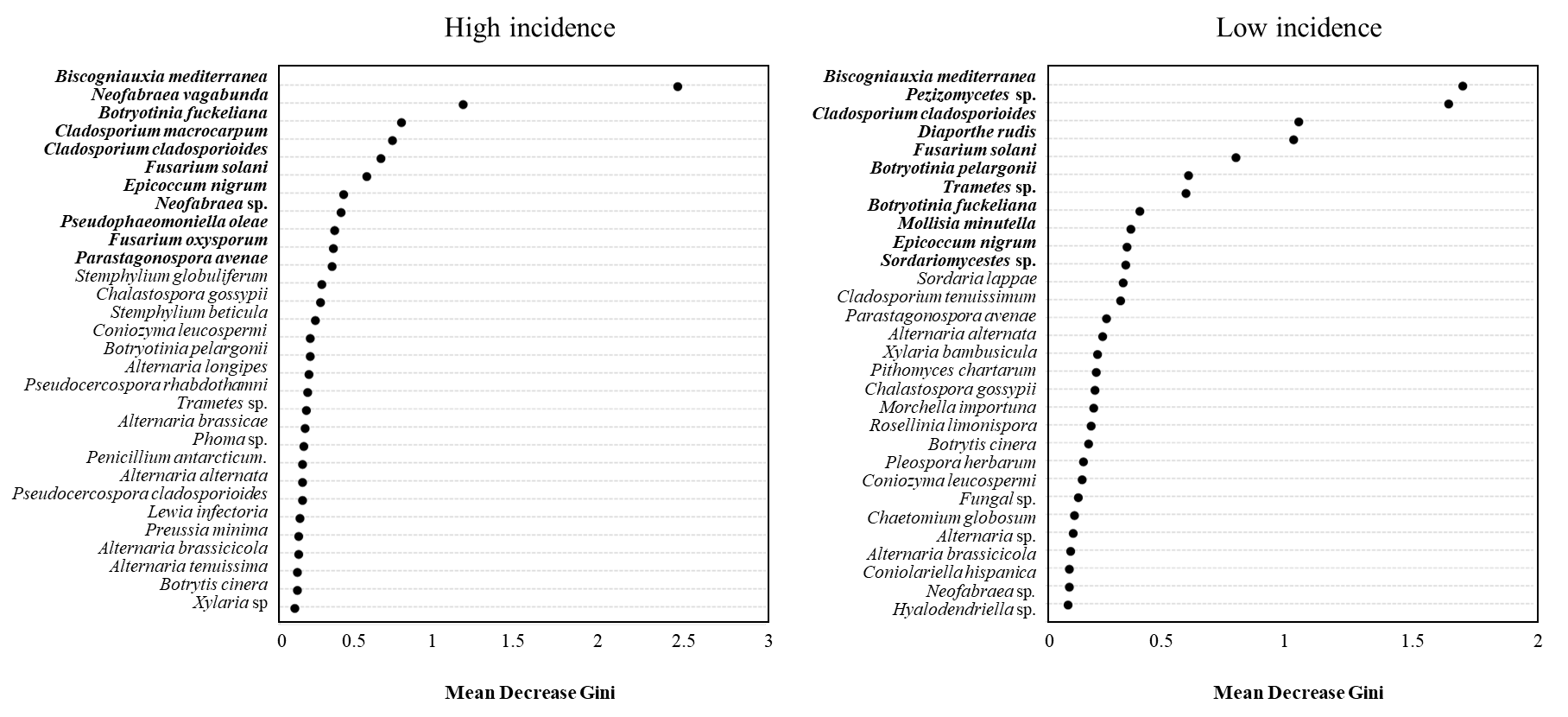
).

## Supplementary Tables

**Table S1**. Correlation coefficient (Cr) between the abundance of endophytic fungal operational taxonomic units preselected by the random forest analysis with the abundance of the pathogen *Colletotrichum godetiae* or *Colletotrichum fioriniae*. All Cr values are significant at p<0.001. A correlation of -1.0 shows a perfect negative correlation, while a correlation of 1.0 shows a perfect positive correlation. A correlation of 0.0 shows no relationship between the two variables. Positive correlations are indicated in bold.

| **Endophytic fungi** | **Pathogenic fungi** | **Correlation coefficient** |
| --- | --- | --- |
| *Biscogniauxia mediterranea* | *Colletotrichum fioriniae* | -0.206 |
| *Botryotinia fuckeliana* | *Colletotrichum fioriniae* | -0.141 |
| *Cladosporium* aff. *cladosporioides* | *Colletotrichum fioriniae* | -0.013 |
| *Cladosporium* aff. *macrocarpum* | *Colletotrichum fioriniae* | -0.108 |
| *Biscogniauxia mediterranea* | *Colletotrichum godetiae* | -0.409 |
| *Botryotinia fuckeliana* | *Colletotrichum godetiae* | -0.153 |
| *Cladosporium* aff. *cladosporioides* | *Colletotrichum godetiae* | -0.466 |
| *Cladosporium* aff. *macrocarpum* | *Colletotrichum godetiae* | -0.458 |
| *Epicoccum nigrum* | *Colletotrichum fioriniae* | -0.141 |
| *Epicoccum nigrum* | *Colletotrichum godetiae* | -0.480 |
| *Fusarium* aff. *oxysporum* | *Colletotrichum fioriniae* | -0.091 |
| *Fusarium* aff. *oxysporum* | *Colletotrichum godetiae* | -0.370 |
| ***Fusarium* aff. *solani*** | ***Colletotrichum fioriniae*** | **0.193** |
| *Fusarium* aff. *solani* | *Colletotrichum godetiae* | -0.109 |
| ***Neofabraea* sp.** | ***Colletotrichum fioriniae*** | **0.501** |
| ***Neofabraea* sp.** | ***Colletotrichum godetiae*** | **0.488** |
| *Neofabraea vagabunda* | *Colletotrichum fioriniae* | -0.135 |
| ***Neofabraea vagabunda*** | ***Colletotrichum godetiae*** | **0.657** |
| ***Parastagonospora avenae*** | ***Colletotrichum fioriniae*** | **0.010** |
| *Parastagonospora avenae* | *Colletotrichum godetiae* | -0.097 |
| ***Pseudophaeomoniella oleae*** | ***Colletotrichum fioriniae*** | **0.810** |
| ***Pseudophaeomoniella oleae*** | ***Colletotrichum godetiae*** | **0.569** |

**References**

1. Ondov, B., Bergman, N. & Phillippy, A. Interactive metagenomic visualization in a Web browser. *BMC Bioinformatics* **12**, 385 (2011)
